# Hepatitis C care delivery practices among buprenorphine prescribers and non-prescribers: results from a survey of Washington state primary care providers

**DOI:** 10.1186/s13722-025-00603-9

**Published:** 2025-09-24

**Authors:** Jocelyn R. James, Amelia M. Mohabir, Claire B. Simon, Allison Cole, Emalie Huriaux, Jon Stockton, Julien Rouvere, Judith I. Tsui

**Affiliations:** 1 Department of Medicine, Division of General Internal Medicine, University of Washington School of Medicine, Harborview Adult Medicine Clinic, 325 9th Ave., Box 359780, Seattle, WA 98104 USA; 2 University of Washington School of Medicine, 1959 NE Pacific St, Seattle, WA 98195 USA; 3 Department of Family Medicine, University of Washington School of Medicine, 4311 11th Ave. NE, Suite 210, Seattle, WA 98105 USA; 4 Washington State Department of Health, 101 Israel Rd SE, Tumwater, WA 98501 USA

**Keywords:** Hepatitis c, Opioid related disorders, Buprenorphine

## Abstract

**Background:**

Hepatitis C infection (HCV) and opioid use disorder (OUD) are syndemic in the U.S., thus primary care providers (PCPs) who treat OUD by prescribing buprenorphine can play key roles to advance HCV elimination targets. We compared HCV screening and treatment among PCPs who do and do not prescribe buprenorphine in Washington (WA) State.

**Methods:**

This study utilized a cross-sectional survey of PCPs in WA State, designed to characterize HCV care delivery practices and experiences/attitudes toward HCV. In this study, the independent variable was self-reported buprenorphine prescribing, and the main outcomes were (1) guideline-concordant HCV screening and (2) directly providing treatment for HCV. We used descriptive statistics to describe respondent characteristics. We used logistic regression to assess the association between buprenorphine prescribing status and HCV screening and treatment outcomes.

**Results:**

Our sample included 73 PCPs, of whom 55% prescribe buprenorphine. We found that 25% of buprenorphine prescribers directly treated HCV. There was over a 2x greater relative odds that buprenorphine prescribers would correctly screen for HCV relative to non-prescribers (OR = 2.24; 95% CI: 0.67–8.18, *p* = .20) and a nearly 2.5x greater relative odds that they would treat HCV relative to non-prescribers (OR = 2.42; 0.72–9.61; *p* = .17), although both findings were not statistically significant.

**Conclusion:**

In a sample of PCPs in WA state, buprenorphine prescribers compared to non-prescribers appear more likely to screen for and directly treat HCV, yet only a minority treat HCV. Interventions are needed to enhance HCV guideline-concordant care among these and all PCPs on the frontlines of caring for persons with OUD.

**Supplementary Information:**

The online version contains supplementary material available at 10.1186/s13722-025-00603-9.

## Introduction

Hepatitis C virus (HCV) is the most common, chronic bloodborne infection in the United States, with an estimated 2.2 million Americans living with HCV as of 2018. Since 2012, HCV has exceeded HIV as a cause of mortality [1]. The incidence of HCV has been increasing in recent decades: the number of acute cases has doubled since 2014 [2]. Increased injection drug use associated with the public health crisis of poorly managed pain, opioid misuse, and opioid use disorder is the primary contributor to this second epidemic of HCV infection, which disproportionately impacts young adults and extends to rural settings [2–4]while the first epidemic primarily impacted people in the “Baby Boomer” cohort (born between 1945 and 1965).

The elimination of the public health threat of HCV, made possible by the development of highly- effective direct acting antivirals (DAA), is a shared goal at the global, national, and state levels. Since 2020, the U.S. Preventive Services Task Force has recommended screening all adults age 18–79 for HCV infection [5]; the Centers for Disease Control and Prevention similarly recommends universal HCV screening for adults as well as screening with each pregnancy and periodic rescreening of persons who inject drugs and share injection equipment [6]. Guidelines recommend that HCV screening comprise HCV antibody testing with reflexive HCV RNA testing. Data from both clinical trials and real-world settings demonstrates that DAA treatment leads to high rates of cure among people who use drugs, including those with recent injection drug use [7]. In 2018, two years after the World Health Organization (WHO) announced a goal of eliminating HCV by 2030 [8], Washington (WA) State’s governor, Jay Inslee, issued a directive to state agencies to eliminate the public health threat of HCV by 2030 [9]. In response to this directive, the WA State Health Care Authority removed remaining barriers to treatment in the state’s Medicaid program to increase HCV treatment access. As of July 1, 2019, WA State Medicaid policy now allows all prescribers, including primary care clinicians, to treat uncomplicated HCV; specialist referral is not required [10]. Additionally, WA state entered into a modified subscription model agreement with the manufacturer of one of the two FDA-approved pan-genotypic DAAs (glecaprevir/pibrentasvir), such that any clinician with prescribing authority can now prescribe DAAs for HCV without prior authorization. Yet even after such policy changes intended to expand HCV treatment access, WA State has not seen dramatic increases in HCV treatment rates [11]; rather, efforts to reach recommended thresholds for treatment and cure fall short in WA as with the rest of the country [12].

Such failures of policy to produce expected results highlight implementation challenges and how multilevel barriers must be addressed to affect change. One challenge is the shortage of HCV treatment providers who interface with persons who use drugs (PWUD), a population that should, according to guidelines, be prioritized for HCV treatment [13]. WHO recently recommended a shift to delivering HCV testing and treatment in primary care, and for care to be delivered by general practitioners and nurses, rather than specialists. Given that opioid use disorder (OUD) and HCV are syndemic conditions [14, 15]more providers are needed who can utilize the tools available to treat both diseases. There has been a major push for PCPs to prescribe buprenorphine [16]an effective medication for OUD that can be offered in an office-based setting. Preparing this workforce to also offer HCV treatment would be ideal. It is unknown to what extent this is happening and whether PCPs who treat OUD are more likely to offer HCV care, including direct treatment with DAAs. Thus, the aim of this study was to characterize HCV screening and treatment practices among PCPs who do and do not prescribe buprenorphine for OUD.

## Methods

### Study design and participants

For the purposes of this study, we utilized a subset of questions from a cross-sectional survey of WA State PCPs designed to characterize HCV care delivery practices and experiences/attitudes toward HCV care delivered by pharmacists. The survey included a convenience sample of WA State PCPs recruited through professional networks in Internal Medicine, Family Medicine, and Addiction Medicine. Surveys were electronically distributed to provider groups, conference attendees, and professional societies via email lists and newsletters in the spring of 2023. A subset of participants from a network of rural serving clinics were offered a $20 gift card for completing the survey to enhance survey responses.

### Survey instrument

The 37-item survey (Appendix 1) was administered via REDCap and included questions on demographics, basic practice characteristics, substance use care, HCV treatment-related views and practices, and views and experiences relating to collaboration with pharmacists and collaborative models of HCV treatment. We included the question, *“Do you currently provide primary care services as part or all of your practice?”* to ensure that our population was composed of PCPs as intended. Forced choice response was not used, so participants were able to complete the survey without answering every question.

### Measures

For purposes of this study, the independent variable was participant self-report of prescribing vs. not prescribing buprenorphine, assessed by the survey question, “*Do you prescribe buprenorphine for treatment of heroin or other opioid addiction?”* Participants could answer “yes” or “no;” those who did not respond were classified as non-prescribers, with the rationale that that those who did not respond may not have been familiar with what buprenorphine is and therefore were unlikely to be prescribers. Primary outcome variables were (1) approach to HCV screening, assessed by the question, “*How do you approach screening for hepatitis C?”* and (2) approach to HCV treatment, assessed by the question “*How do you approach treatment of hepatitis C among your patients?”* Participants were asked to choose the best fit answer from a list of responses. Secondary outcome variables included (1) offering HCV treatment to patients with recent illicit drug use, assessed by the question, “*Do you offer hepatitis C treatment to people with illicit drug use within 90 days?”* and (2) offering HCV treatment to people with problematic alcohol use and current drinking, assessed by the question, “*Do you offer hepatitis C treatment to people with alcohol use disorder or unhealthy drinking who currently drink?”* For both questions, response options included “never,” “some of the time,” “most of the time,” “always,” “decline to answer,” and “NA.”

Descriptive variables included age, gender, race/ethnicity, provider type (physician, nurse practitioner, physician assistant, other), resident/fellow status, practice type (community health clinic, private office practice, hospital associated clinic, health maintenance organization, academic/university clinic, federally operated clinic (e.g. VA), other), most common patient insurance (Medicaid, Medicare, private, other), and estimate of percentage of patients seen who inject drugs.

### Statistical analysis

Descriptive statistics were used to characterize the survey population, including buprenorphine prescribers and non-prescribers. Fisher’s exact tests were used to compare demographic characteristics between the two groups. Logistic regression models were used to examine associations between buprenorphine prescribing status and guideline-concordant HCV screening and directly treating HCV. Differences between prescribers and non-prescribers in offering HCV treatment to people with recent illicit drug use and to those with AUD or unhealthy drinking were examined via Fisher’s exact tests, with outcomes dichotomized to compare participants who endorsed ‘always’ to those who endorsed ‘never,’ ‘some of the time’, or ‘most of the time.’ Adjusted analyses were not performed due to sample size limitations and lack of prior compelling evidence supporting certain factors as major confounders. Analyses were conducted in R Version 4.3.2 [17], and a threshold of *p* < .05 was used for hypothesis testing.

## Results

Demographic and general practice characteristics of the study sample by bupreprenorphine prescribing status are presented in Table 1. Prescribers and non-prescribers of buprenorphine were similar with the exception that buprenorphine prescribers reported having a higher proportion of patients who were Medicaid insured (68% v. 21%; *p* = .001).

**Table 1:** Participating provider demographics and practice characteristics among those who provide primary care services as part or all of their practice. (*N* = 73)

Provider demographics and practice characteristics	Prescribes buprenorphine, *n* = 40 (55%)	Does not prescribe buprenorphine, *n* = 33 (45%)	*p*-value^a^
Age (years), mean (SD)	43.6 (10.7)	46.5 (15.8)	0.45
Gender^b^			
Woman	27 (68%)	21 (64%)	0.78
Man	13 (33%)	10 (30%)	
Prefer not to identify	0	1 (3%)	
Missing	0	1 (3%)	
Race			
Black or African American	1 (3%)	0	0.20
Asian	5 (13%)	4 (12%)	
White	31 (78%)	23 (70%)	
More than one	2 (5%)	1 (3%)	
Other^c^	0	2 (6%)	
Prefer not to identify	0	3 (9%)	
Missing	1 (3%)	0	
Ethnicity			
Hispanic/Latino	3 (8%)	3 (9%)	0.27
Non-Hispanic/Latino	35 (88%)	26 (79%)	
Prefer not to identify	1 (3%)	4 (12%)	
Missing	1 (3%)	0	
Provider type			
Physician	33 (83%)	24 (73%)	0.31
Nurse Practitioner	3 (8%)	6 (18%)	
Physician Assistant	4 (10%)	2 (6%)	
Other^d^	0	1 (3%)	
Missing	0	0	
If you indicated being a physician, are you a trainee (resident or fellow)?^e^			
No	22 (55%)	20 (61%)	0.16
Yes	8 (20%)	2 (6%)	
Missing	3 (8%)	2 (6%)	
Practice clinic/facility type			
Community health clinic	4 (10%)	1 (3%)	0.47
Private office practice or freestanding clinic	3 (8%)	6 (18%)	
Hospital-associated clinic	18 (45%)	12 (36%)	
Health maintenance organizatino or other prepaid practice (e.g., Kaiser)	0	1 (3%)	
Community health center (e.g., Federally Qualified Health Center)	3 (8%)	1 (3%)	
Academic/university-affiliated clinic	11 (28%)	9 (27%)	
Federal government-operated clinic (e.g., VA)	1 (3%)	2 (6%)	
Other^b^	0	1 (3%)	
Missing	0	0	
Most common insurance type among patients			
Medicaid	27 (68%)	7 (21%)	0.001
Medicare	6 (15%)	8 (24%)	
Private insurance	3 (8%)	9 (27%)	
None of the above/unable to estimate	4 (10%)	9 (27%)	
Missing	0	0	
County of primary care practice^f^			
Rural	7 (18%)	3 (9%)	0.22
Urban	31 (78%)	21 (64%)	
Don’t know	1 (3%)	4 (12%)	
Missing	1 (3%)	5 (15%)	
In your best estimate, what percentage of your patients have a history of injecting drugs?			
Less than or equal to 25%	28 (70%)	28 (85%)	0.16
Greater than 25%	9 (23%)	2 (6%)	
Unable to estimate	3 (8%)	2 (6%)	
Missing	0	1 (3%)	

^a^The *p*-value for age was computed via an independent samples *t*-test; remaining *p*-values were computed via Fisher’s exact tests

^b^“Woman” and “man” were asked as “female” and “male,” respectively

^c^No participants who selected ‘Other’ for race or practice clinic/facility type provided write-in responses

^d^One participant who selected ‘Other’ indicated that they are a pharmacist

^e^This item was only shown to participants who indicated that they are physicians; percentages are calculated out of column totals

^f^Urban Influence Codes were used to determine rurality based on county of primary care practice

The percentage of participants performing guideline-concordant screening for HCV was greater among buprenorphine prescribers compared to non-prescribers, though this difference was not significant (35/40 or 88% vs. 25/33 or 76%, *p* = .23; Table 2). Similarly, more buprenorphine prescribers compared to non-prescribers reported prescribing HCV treatment for their patients (10/40 or 25% vs. 4/33 or 12%, *p* = .23), though this finding also did not attain statistical significance. Buprenorphine prescribers were no more likely than non-prescribers to “always” offer HCV treatment to those with problematic drinking and current alcohol use (14/26 or 54% vs. 5/20 or 25%, *p* = .07) or “always” offer HCV treatment to patients with illicit drug use within 90 days (15/26 or 58% vs. 5/17 or 29%, *p* = .12). While neither finding was statistically significant, we found a two-fold greater relative odds that buprenorphine prescribers will appropriately screen for HCV compared to non-prescribers (unadjusted OR [95% CI] = 2.24 [0.67–8.18], *p* = .20) and a two-fold greater relative odds that buprenorphine prescribers will treat HCV relative to non-prescribers (unadjusted OR [95% CI] = 2.42 [0.72–9.61], *p* = .17, data not shown).

**Table 2:** HCV screening and treatment characteristics among providers who provide primary care services as part of their practice, shown by those who do vs. do not prescribe buprenorphine. (*N* = 73)

Provider HCV screening and treatment characteristics	Prescribes buprenorphine, *n* = 40 (55%)	Does not prescribe buprenorphine, *n* = 33 (45%)	Total, *n* (%)	*p*-value^a^
Do you approach for hepatitis C screening all patients 18 years or older or those aged 18–79?				0.23
No	5 (13%)	8 (24%)	13 (18%)	–^b^
Yes	35 (88%)	25 (76%)	60 (82%)	–
Do you prescribe hepatitis C treatment among your patients?				0.23
No	30 (75%)	29 (88%)	59 (81%)	–
Yes	10 (25%)	4 (12%)	14 (19%)	–
Do you offer hepatitis C treatment to people with illicit drug use within 90 days? (*n* = 43)^c^				0.12^d^
Never	4 (15%)	6 (35%)	10 (23%)	–
Some of the time	4 (15%)	4 (24%)	8 (19%)	–
Most of the time	3 (12%)	2 (12%)	5 (12%)	–
Always	15 (58%)	5 (29%)	20 (47%)	–
Do you offer hepatitis C treatment to people with alcohol use disorder or unhealthy drinking who currently drink? (*n* = 46)^c^				0.07^d^
Never	3 (12%)	10 (50%)	13 (28%)	–
Some of the time	3 (12%)	3 (15%)	6 (13%)	–
Most of the time	6 (23%)	2 (10%)	8 (17%)	–
Always	14 (54%)	5 (25%)	19 (41%)	–

^a^Fisher’s exact tests *p*-values

^b^Not applicable

^c^Total excludes participants who selected ‘Decline to answer’ or ‘NA’; percentages are calculated out of the total number of available responses

^d^Fisher’s exact test *p*-value comparing participants who endorsed ‘always’ to those who endorsed ‘never,’ ‘some of the time,’ or ‘most of the time.’

## Discussion

This study of PCPs in WA State found that providers who were buprenorphine prescribers were more likely to provide guideline-concordant HCV care compared to PCPs who were not buprenorphine prescribers, although these differences were not statistically significant. Even among buprenorphine prescribers, only 1 in 4 reported directly offering HCV treatment. These results suggest there is need and opportunity to enhance HCV guideline-concordant screening and treatment among PCPs on the frontline of caring for PWUD.

Despite having the tools to cure nearly all individuals with HCV for more than a decade, the U.S. is greatly falling short of HCV elimination goals. WA State is no exception, despite being on the forefront of policy changes to facilitate access to DAAs. The uptick that was seen over several years in HCV incidence in WA State, particularly among young adults, has the potential to overwhelm elimination efforts and may lead to substantial future morbidity and mortality if unaddressed [18]. The experience of the Veterans Health Administration, which has overseen increases in the percentage of patients with HCV receiving treatment and attaining cure from 17% to 7%, respectively (pre -DAAs), to 80.1% and 91.6%, respectively (post-DAAs), demonstrates the vast promise of DAAs [19]. Evidence strongly supports equivalent DAA cure rates among PCPs and other non-specialist providers, and task-shifting is a global recommendation. Buprenorphine prescribers are on the leading edge of caring for those patients at risk for HCV infection and thus are well-positioned to lead elimination efforts. Our findings are encouraging, as they suggest that among PCPs in WA State, buprenorphine prescribers may already be more educated and willing to provide HCV care, including treatment. The finding that many do not routinely offer HCV treatment to patients with current illicit drug use or unhealthy drinking is of concern. Evidence shows that persons with active alcohol and substance use can have nearly equal HCV cure rates [20]and guidelines do not endorse withholding DAAs for patients with active substance use [21]. While our study was not designed to explore system barriers to HCV treatment, it did include one question asking respondents who do not treat HCV why this was so; lack of training/time to train and perceived lack of need were identified as the main barriers [22]. Clearly, more extensive work is needed to identify and understand the persistent barriers that stand in the way of all PCPs offering guideline concordant HCV care.

Several limitations of this study may have affected the results. The sample size was modest and thus analyses may not have been sufficiently powered to detect significant differences between the groups. There were missing responses for the survey question assessing whether participants prescribed HCV treatment to persons with recent drug or alcohol use; this could relate to reluctance to truthfully disclose (social desirability bias) or to lack of clarity in interpreting the questions (e.g., whether the time frame of 90 days referred to drug use or the window for offering HCV treatment). No cognitive testing was performed on the survey questions to ensure question comprehension. It is possible that offering compensation to participants in smaller networks but not those in larger networks could have biased the composition of our sample. Given that our survey was exclusively administered to PCPs in WA State, results reported here may not be generalizable to other states and regions.

In summary, among a sample of PCPs in WA State who were a mix of prescribers and non-prescribers of buprenorphine, we found that although our findings did not reach statistical significance, those who prescribe buprenorphine were more likely to screen and treat patients for HCV. While it is important to support PCPs in general to treat HCV, efforts should be made to specifically support PCPs who are buprenorphine prescribers to sustain and enhance their activities in providing HCV screening and treatment, to reach elimination goals.

## Supplementary Information

Below is the link to the electronic supplementary material.

Supplementary Material 1

## Data Availability

Data will be accessible through requests made to the first and/or senior author.
