# Supplementary material for: Hepatitis C care delivery practices among buprenorphine prescribers and non-prescribers: results from a survey of Washington state primary care providers
Source: Addict Sci Clin Pract. 2025 Sep 24;20:75. doi: 10.1186/s13722-025-00603-9 (PMC12462307; doi:10.1186/s13722-025-00603-9)
Supplement: Supplementary file 1 — Supplementary Material 1 [file 13722_2025_603_MOESM1_ESM.docx]

| **Topic** | **Response** |
| --- | --- |
| Demographics | |
| 1. What is your age? | - ________ |
| 1. What is your gender? | - Female - Male - Non-binary/other - Prefer not to identify |
| 1. What is your race? | - Black or African American - Asian - Native Hawaiian or Other Pacific Islander - American Indian or Alaskan Native - White - More than one - Other - Prefer not to identify |
| 1. If other, specify: | |
| 1. What is your ethnicity? | - Hispanic/Latino - Non- Hispanic/Latino - Prefer not to identify |
| Basic practice characteristics | |
| 1. What type of provider are you? | - Physician [ branch to are you a trainee (resident, fellow)? y/n] - Nurse Practitioner - Physician Assistant - Other |
| 1. If other, specify: | |
| 1. Do you currently provide primary care services as part or all of your practice? | - Yes - No |
| 1. What type of clinic/facility do you work in? Choose single best-fit answer. | - Community health clinic - Private office practice or freestanding clinic - Hospital-associated clinic - Health maintenance organization or other prepaid practice (e.g., Kaiser) - Community Health Center (e.g., Federally Qualified Health Center (FQHC)) - Non-federal government clinic (e.g., state, county, city, maternal and child health, etc.) - Academic /university-affiliated clinic - Federal government -operated clinic (e.g., Veterans’ Administration, military) - Tribal health center/Indian Health Service/Clinic - Other |
| 1. If other, specify: | |
| 1. In your best estimate, what insurance coverage is most common for your patients? | - Medicaid - Medicare - Private insurance - None of the above/ unable to estimate |
| 1. In which county is your primary care practice? | - Drop-down list of 39 counties - Don’t know |
| 1. What is the zip code of your primary care practice? | - _________ - Don’t know |
| Substance use | |
| 1. Do you prescribe buprenorphine for treatment of heroin or other opioid addiction? | - Yes - No |
| 1. In your best estimate, what percentage of your patients have a history of injecting drugs? | - Less than 1% - 1% to 25% - Greater than 25% and up to 50% - Greater than 50% and up to 75% - Greater than 75% - Unable to estimate |
| Hepatitis C | |
| 1. Do providers in your clinic treat hepatitis C infection (i.e., prescribe medication for hepatitis C)? | - Yes - No: SKIP to 16 |
| 1. Which providers in your clinic treat hepatitis C infection? Select all that apply. | - Physicians - Nurse Practitioners - Physician Assistants - Other |
| 1. If other, specify: | |
| 1. Which clinical team members in your clinic collaborate with providers to treat hepatitis C infection? Select all that apply. | - Nurses - Pharmacists - Social workers - Other |
| 1. If other, specify: | |
| 1. Where do providers in your clinic refer patients for hepatitis C treatment? Select all that apply. | - Other providers in the clinic/health system   [branch to infectious disease specialist, gastroenterology/hepatology specialist, other non-specialist provider]  [for each response, branch to on-site or off-site]   - Outside the clinic/health system   [branch to infectious disease specialist, gastroenterology/hepatology specialist, other non-specialist provider]   - Providers in my clinic do not refer patients for hepatitis C treatment - Don’t know |
| 1. How do you approach screening for hepatitis C? Select all that apply. | - Screen all 18 years or older or those from age 18-79 - Screen those born 1945-1965 - Screen those with abnormal liver tests and/or symptoms - Screen based on risk factors - I do not screen - Other |
| 1. If other, specify: | |
| 1. Among your patients, what proportion of those with hepatitis C infection have been seen by a provider for hepatitis C treatment? Please provide your best estimate. | - Less than 1% - 1% to 25% - Greater than 25% and up to 50% - Greater than 50% and up to 75% - Greater than 75% - Unable to estimate |
| 1. Among your patients, what proportion of those with a history of hepatitis C infection have been cured? Please provide your best estimate. | - Less than 1% - 1% to 25% - Greater than 25% and up to 50% - Greater than 50% and up to 75% - Greater than 75% - Unable to estimate |
| 1. How do you approach treatment of hepatitis C among your patients? Select all that apply. | - I prescribe hepatitis C treatment - I do NOT prescribe hepatitis C treatment - I refer for treatment - Other |
| 1. If other, specify: | |
| 1. If you do NOT prescribe hepatitis C treatment, why is this? Select all that apply. | - None or few of my patients have hepatitis C (i.e., not enough volume) - Others in my practice /health system offer hepatitis C treatment (not enough need) - Not interested in providing hepatitis C treatment - Not enough time to learn how to treat hepatitis C - Not enough time to deliver hepatitis C care - Not trained to provide hepatitis C treatment - Concerned that I’m not authorized by payors to prescribe hepatitis C treatment - Not authorized by clinic/ health system to prescribe hepatitis C treatment - Clinic does not have support in place to treat hepatitis C - Need help with prior authorization - No pharmacies in my area will fill prescription for hepatitis C treatment - Don’t know where to send prescription (where patient can fill prescription) - Other |
| 1. If other, specify: | |
| 1. Estimate the number of patients you have treated for hepatitis C in the last 2 years. | - 0 - 1-20 - 21-50 - >50 - NA |
| 1. Do you offer hepatitis C treatment to people with illicit drug use within 90 days? | - Never - Some of the time - Most of the time - Always - Decline to answer - NA |
| 1. Do you offer hepatitis C treatment to people with alcohol use disorder or unhealthy drinking who currently drink? | - Never - Sometimes - Most of the time - Always - Decline to answer - NA |
| 1. What trainings/ supports have you utilized when treating hepatitis C? Select all that apply. | - Online resources or tutorials such as Up To Date, hcvguidelines.org (IDSA/AASLD guideline), etc. - Printed educational materials - Webinars - Project ECHO - Consultation with pharmacist   [branch: within organization, community-based, other]   - Consultation with colleague /specialist   [branch: within organization, outside organization]   - Collaborative practice with pharmacist - Collaborative care model with nurses - Other - None - NA |
| 1. If other, specify: | |
| Collaborative care with pharmacists | |
| 1. Is there at least one clinical pharmacist embedded within your clinic? | - Yes (SKIP to 28) - No |
| 1. Do you have access to clinical pharmacists within your health system? | - Yes - No - Unsure |
| 1. Which of the following best describes the frequency of your previous collaboration with pharmacists? | - I have never rarely/never collaborated with pharmacists in the past - I have sometimes collaborated with pharmacists in the past - I have frequently collaborated with pharmacists in the past - I have always or nearly always collaborated with pharmacists in the past |
| 1. Which of the following describe the nature of your communication/ collaboration with pharmacists in the routine practice of patient care? Select all that apply. | - Limited and/or brief communication, e.g. clarifying dispensing instructions or other prescribing information   [branch, all that apply: within organization, outside organization]   - Consultation with clinical pharmacists about specific patient care questions [branch, all that apply: within organization, outside organization] - Collaboration or co-management of patients with chronic medical conditions such as hypertension, diabetes, etc.   [branch, all that apply: within organization, outside organization]   - I do not communicate or collaborate with pharmacists - Other |
| 1. If other, specify: | |
| 1. In the State of WA, pharmacists (in partnership with physicians) can help with management of numerous medical conditions through "Collaborative Drug Therapy Agreements (CDTAs)". Are you familiar with this care delivery model in general? | - Yes - No - Unsure |
| 1. Does your practice currently utilize CDTAs to deliver care for any medical conditions? | - Yes - No - Unsure |
| 1. For which conditions does your practice currently utilize CDTAs with pharmacists? Select all that apply. | - Hypertension - Diabetes - Heart failure - Chronic kidney disease - COPD - HIV - Other |
| 1. If other, specify: | |
| 1. Does your practice currently utilize pharmacists/CDTAs to deliver care for hepatitis C? | - Yes (SKIP to 36) - No - Unsure |
| 1. Would you be interested in establishing a CDTA with a pharmacist to provide hepatitis C treatment to your patients? | - Yes - No - Unsure - Decline to answer |
| 1. Would you be interested in referring patients to a pharmacist who has a CDTA with another physician for hepatitis C treatment? | - Yes - No - Unsure - Decline to answer |
| 1. What concerns do you have, if any, about delivery of hepatitis C care by pharmacists practicing under a CDTA? Select all that apply. | - No concerns - Lack of time to coordinate care - Lack of compensation for provider - Care coordination and/or fragmentation - Risk management - Other |
| 1. If other, specify: | |
| 1. Assuming that protocols were in place, how comfortable would you be with pharmacists in your practice assisting with hepatitis C treatment in the following ways, rated from 1 (extremely uncomfortable) to 5 (extremely comfortable)?   Gathering hepatitis C targeted history (e.g., duration of infection, history of prior treatment, alcohol use)    Ordering/interpreting tests to diagnose hepatitis C    Ordering/interpreting pre-treatment tests (hepatitis B, HIV, basic labs, calculated fibrosis scores)    Reviewing medication interactions    Identifying patients who merit referral to a specialist    Making treatment recommendations    Dispensing medications and ensuring pick-up of refills    Counseling/education of patients starting treatment (e.g., how to take the medication, potential side effects, etc.)    Monitoring patients on treatment, as needed  Responding to side effects of treatment    Assisting with care coordination prior to and during treatment | - 1 (extremely uncomfortable) - 2 (mildly uncomfortable) - 3 (neutral) - 4 (comfortable) - 5 (extremely comfortable) |
